# Mechanisms by which SIRT2 modulates alveolar macrophage immunoreactivity to intervene in *Actinobacillus pleuropneumoniae* infection in piglets under cold stimulation

**DOI:** 10.1186/s13567-026-01780-4

**Published:** 2026-06-14

**Authors:** Rongge Xia, Wanqun Xing, Zhiqi Zhu, Qi Han, Xinpeng Chen, Huiying Shi, Wencong Wu, Boxi Zhang, Jingjing Lu, Wenjing Guo, Lan He, Bin Xu

**Affiliations:** 1 College of Animal Science and Veterinary Medicine, Heilongjiang Bayi Agricultural University, Daqing, 163319 Heilongjiang China; 2 State Key Laboratory of Agricultural Microbiology, College of Veterinary Medicine, Huazhong Agricultural University, Wuhan, 430070 Hubei China; 3 Hubei Jiangxia Laboratory, Wuhan, 430200 Hubei China; 4 College of Animal Science and Veterinary Medicine, Jilin University, Changchun, 130000 Jilin China

**Keywords:** *Actinobacillus pleuropneumoniae* (APP), immune response, porcine alveolar macrophages (PAMs), silent information regulator 2 (SIRT2), acetylation

## Abstract

*Actinobacillus pleuropneumoniae* (APP) is a porcine respiratory pathogen that causes significant economic losses to the global swine industry. APP-induced piglet respiratory disease complex (PRDC) is highly prevalent in winter and triggers an intense inflammatory response in the lungs at disease onset, accompanied by severe infiltration of porcine alveolar macrophages (PAMs). The magnitude of the PAM-mediated immune response following APP infection is a key factor influencing the regression of respiratory disease. However, it remains unclear whether cold stimulation exacerbates this response. In this study, we found that cold stimulation exacerbated APP-induced lung injury and enhanced macrophage activation. Metabolomics and transcriptomics analyses revealed that immune cells underwent metabolic reprogramming in response to energy mobilisation after APP infection, thereby interfering with lung immune function. In addition, we identified silent information regulator 2 (SIRT2) as a key regulator of PAM activation and demonstrated at the molecular level that SIRT2 mediates the immune response to PAMs following APP infection by regulating the acetylation modification of NF-κB p65. In conclusion, our results elucidate the role of SIRT2 in regulating the immune response to PAMs in piglets infected with APP under low-temperature conditions. These findings may provide potential intervention targets and effective control pathways to address the overactivity of immune cells induced by environmental stress and reduce the high incidence of winter respiratory disease in piglets.

## Introduction

Low ambient temperatures are a significant contributing factor to the high incidence and severity of porcine respiratory disease complex (PRDC), and to the challenges in its prevention and control during autumn and winter [1]. Young animals have low tolerance and poor environmental adaptability, leading to a marked increase in primary and secondary respiratory diseases when they are exposed to low temperatures [2]. The scientific prevention and control measures implemented in intensive pig farms have significantly reduced the occurrence of large-scale epidemics caused by virulent respiratory pathogens, such as porcine reproductive and respiratory syndrome virus (PRRSV). However, PRDC caused by conditionally pathogenic microorganisms, including *Actinobacillus pleuropneumoniae* (APP), still remains high in prevalence during winter, with low-temperature-triggered epidemics resulting in growth retardation, increased mortality in piglets, and higher biocontainment costs [3]. Macrophage dysfunction is a key factor influencing host immunity under low-temperature conditions [4]. Porcine alveolar macrophages (PAMs), the sentinel cells of the respiratory system, defend against foreign pathogens and maintain host health through homeostasis. PAMs mediate defence and antigen presentation, initiating immune responses induced by changes in microenvironmental cues [5].

Overactivation of PAMs frequently occurs after piglets are infected with APP and other pathogens, compromising the barrier function of the host’s innate immune system. This results in more severe lung lesions, the potential development of a cytokine storm, and increased susceptibility to secondary infections and mortality in piglets [6]. Therefore, systematically elucidating the mechanism underlying excessive immune responses in PAMs under low-temperature conditions is crucial for preventing the onset and progression of PRDC during winter, safeguarding piglet health, and ensuring the stability of the swine industry.

Metabolic adaptation is a defining feature and prerequisite for PAM activation [7], which is highly energy-consuming. As such, metabolic reprogramming frequently occurs during this process to meet the increased energy demands. During this process, cellular energy uptake shifts towards the Warburg effect, transitioning from glucose oxidative phosphorylation to aerobic glycolysis to ensure a rapid supply of ATP [8]. An immunometabolic perspective, therefore, offers new insights and a deeper understanding of immune responses in PAMs. Metabolism-related epigenetic modifications also play a crucial role in regulating immune cell function, exerting diverse bio-regulatory effects by influencing protein activity, localisation, and interactions with other bioactive substances [9]. Preliminary laboratory studies suggest a correlation between cold stimulation and macrophage fate [10]. However, given the characteristics of the macrophage immune response post-APP infection, it remains unclear whether cold stimulation amplifies PAM immune response following APP infection.

In this study, we adopted an immunometabolic approach to investigate how cold stimulation, as a major environmental factor, affects APP-induced lung injury and to explore the mechanisms related to the intervention of PAM immunoreactivity during APP infection under low-temperature conditions. Our results indicate that cold stimulation triggers metabolic microenvironmental changes during energy mobilisation that are critical for regulating the intensity of PAM immunity. Furthermore, silent information regulator 2 (SIRT2) mediates PAM immune responses following APP infection by modulating the acetylation modification of NF-κB p65. These findings provide a theoretical basis for understanding the high incidence and severity of respiratory diseases in piglets during winter and offer new insight and perspectives for preventing and controlling the occurrence and progression of cytokine-storm-like pathology arising from excessive immune responses.

## Materials and methods

### Bacterial strains and culture conditions

*Actinobacillus pleuropneumoniae* (APP) serotype I strain (CVCC259) was kindly donated by Prof Na Li (College of Animal Science and Veterinary Medicine, Jilin University, China) and was preserved in our laboratory. Strains were cultured in brain–heart infusion (BHI) broth medium supplemented with 10% horse serum (Solarbio, S9050) and 10 μg/mL NAD (Solarbio, N8110) at 37 °C.

### Animal experiments

The animal experiments complied with the National Research Council’s *Guide for the Care and Use of Laboratory Animals* (8th edition). The College of Animal Science and Veterinary Medicine, Heilongjiang Bayi Agricultural University, approved the animal breeding and experimental procedures (Permit Number: DWKJXY2024097).

Healthy, unimmunised male weaned Large White piglets (8 ± 0.5 kg) were purchased from Hengtai Pig Farm (Daqing, China) and tested negative for APP antibodies. C57BL/6 mice and *Sirt2*^*−*/*−*^ mice (male, 4–6 weeks old, 20 ± 2 g) were also used in this study. *Sirt2*^*−*/*−*^ mice were generated using CRISPR-Cas9 technology and purchased from Cyagen Biosciences. All animals were housed in individual cages with a 12 h/day light/dark cycle, at 22 ± 1 °C, with free access to food and water. After 1 week of acclimatisation, animals were randomly assigned to experimental groups (Control, Cold, APP, Cold + APP).

For infection experiments, animals were infected intranasally with either 2 × 10^9^ CFU/mL APP (3 mL/piglet) or 1 × 10^8^ CFU/mL APP (100 µL/mouse). Based on the cold exposure model developed in our laboratory, piglets were then subjected to continuous cold stimulation for 6 h in a 4 °C artificial climate chamber [11–13]. This duration was selected based on previous research [14]. For the Cold + APP group, piglets were cold-stimulated after APP infection. At the end of the experiments, animals were anaesthetised with 1.5% pentobarbital sodium and euthanised for blood and tissue collection.

### Routine blood examination

After the cold-stimulated period, piglets were immediately anaesthetised. Blood from the anterior vena cava was collected into an anticoagulant tube with ethylene diamine tetraacetic acid (EDTA)-lithium and analysed using a blood routine examination analyser (IDEXX, USA).

### Serum and tissue biochemical assessment

An additional 2 mL of non-anticoagulated blood was collected and centrifuged to obtain serum. Biochemical parameters were measured according to the manufacturers’ instructions for the malondialdehyde (MDA; Beyotime Biotechnology, S0131S), glutathione peroxidase (GSH-Px; Beyotime Biotechnology, S0056), catalase (CAT; Beyotime Biotechnology, S0051), superoxide dismutase (SOD; Beyotime Biotechnology, S0101S), and lactate dehydrogenase (LDH; Solarbio, BC0680) kits.

### Haematoxylin-eosin (HE) staining

Lung tissues were immersed in 4% paraformaldehyde at 4 °C for 24 h and then processed into paraffin-embedded tissues. Slices (5 µm thick) were cut using a microtome (CM 2016, Germany). The slices were dewaxed with xylene and alcohol, hydrated with alcohol, stained with HE for 5 min, then dehydrated, treated with xylene to enhance transparency, and sealed with resin. Histopathological changes were observed by optical microscopy.

### Masson staining

Sections were immersed in mordant solution and incubated at 60 ℃ for 1 h, then rinsed sequentially with tap water and distilled water. Haematoxylin-eosin (HE) staining solution was injected dropwise for 3 min, and the sections were washed twice with distilled water for 15 s each time. Differentiation was performed in acidic ethanol for several seconds until the tissue appeared completely red, then thoroughly rinsed. Next, 0.1–1% lithium carbonate was added dropwise for 5 min to increase the degree of anti-blue, and then washed with water. Lichun red-pinellia staining solution was added for 10 min and then washed with 2% aqueous glacial acetic acid for 1 min. Phosphomolybdic acid solution was added for approximately 10 min, after which the upper solution was removed, and aniline blue staining solution was added directly for 5 min. Finally, the tissue sections were washed with 0.2% aqueous glacial acetic acid for 1 min, dehydrated with 95% ethanol for 30 s, dehydrated twice with anhydrous ethanol, treated with xylene to induce transparency, and sealed with neutral glue.

### Immunofluorescence staining

The tissue sections were dewaxed with xylene, dehydrated with gradient ethanol (100%–75%), and washed in phosphate-buffered saline (PBS) for 5 min. The dehydrated sections were then placed in EDTA antigen-retrieval buffer for 8 min at a sub-boiling temperature. Subsequently, the sections were blocked in 3% bovine serum albumin (BSA; Sigma-Aldrich, USA). After washing three times with PBS for 3 min, the sections were incubated with a primary antibody overnight at 4 °C. The following day, the corresponding secondary antibody was added and incubated at 37 °C for 50 min. 4′, 6-diamidino-2-phenylindole (DAPI) was used to label the cell nuclei, and the tissue sections were finally mounted with antifade mounting medium.

### Quantitative real-time (qRT)-PCR analysis

Total RNA from cells or tissues was extracted using the TRIzol RNA isolation system (Invitrogen, 15596026) and converted to cDNA using the PrimeScript RT Reagent Kit (TaKaRa, RR037A), followed by the TB Green^®^ Premix Ex Taq^™^ Kit (TaKaRa, RR420A). mRNA levels were calculated using the 2^− ΔΔCt^ method and normalised to β-actin. The primers are listed in Table 1.

**Table 1:** **List of primers used in the RT-qPCR analysis**

Primer name	Gene ID	Sequence (5ʹ–3ʹ)
s*IL-6*-F	399500	GGAGACCTGCTTGATGAGAAT
s*IL-6*-R		CAGCCTCGACATTTCCCTTAT
s*IL-1β*-F	396565	CAAATGCACTTCTGGTGTGGG
s*IL-1β*-R		GCCTTCACGGGTGATTTTCC
s*TNF-α*-F	397086	GCCCTTCCACCAACGTTTTC
s*TNF-α*-R		CAAGGGCTCTTGATGGCAGA
s*CD11b*-F	397459	GCTTTTCCAGGTTCTACGCAC
s*CD11b*-R		TGACTTAAGGCCAAGGCTGT
s*AIF-1*-F	397271	CAGCATCGGCTGAGCTATGA
s*AIF-1*-R		GGACAGGTCCTCATCACTGC
s*SIRT2*-F	100125964	AGTTCTCTGCCTTGTCTCGG
s*SIRT2*-R		ATCGGAGTCCTGAGCCTCTT
s*β-actin*-F	414396	GCAAATGCTTCTAGGCGGAC
s*β-actin*-R		GCGTCCATCACAGCTTCTCA
m*IL-6*-F	16193	CTTCTTGGGACTGATGCTGGTGAC
m*IL-6*-R		TCTGTTGGGAGTGGTATCCTCTGTG
m*IL-1β*-F	16176	CACTACAGGCTCCGAGATGAACAAC
m*IL-1β*-R		TGTCGTTGCTTGGTTCTCCTTGTAC
m*TNF-α*-F	21926	GCGACGTGGAACTGGCAGAAG
m*TNF-α*-R		GCCACAAGCAGGAATGAGAAGAGG
m*CD11b*-F	16409	AGCTTGGCTTTTTCAAGCGG
m*CD11b*-R		AAAGGCCGTTACTGAGGTGG
m*IBA1*-F	11629	TGAGGAGATTTCAACAGAAGCTGA
m*IBA1*-R		CCTCAGACGCTGGTTGTCTT
m*β-actin*-F	11461	TATGCTCTCCCTCACGCCATCC
m*β-actin*-R		GTCACGCACGATTTCCCTCTCAG

*s* refers to *Sus scrofa*, and *m* refers to mouse.

### Western blot analysis

Cells or tissues were lysed in the radioimmunoprecipitation assay (RIPA) lysis buffer (Beyotime Biotechnology, P0013B). Protein concentrations were determined using a bicinchoninic acid (BCA) protein assay kit (Beyotime Biotechnology, P0010S). The proteins were separated through electrophoresis by 8–15% SDS-PAGE gels, then transferred to polyvinylidene difluoride (PVDF) membrane (Millipore, ISEQ00010). The membrane was then incubated in blocking buffer (5% non-fat milk in phosphate-buffered saline with Tween-20 (PBST)) for 2 h at room temperature, washed with PBST, and probed with the corresponding primary antibodies against IL-6 (Proteintech, 66146-1-Ig), IL-1β (Proteintech, 16806-1-AP), TNF-α (Proteintech, 17590-1-AP), HSP60 (Proteintech, 15282-1-AP), HSP70 (Proteintech, 10,995-1-AP), HSP90 (Proteintech, 13171-1-AP), CAT (Proteintech, 21260-1-AP), SOD1 (Cell Signalling, #373855), Keap1 (Proteintech, 80744-1-RR), Nrf2 (Cell Signalling Technology, #12721), HO-1 (Proteintech, 10701-1-AP), SIRT1 (Proteintech, 13161-1-AP), SIRT2 (Proteintech, 66410-1-Ig), NF-κB p65 (Proteintech, 10745-1-AP), Acetyl-NF-κB p65 (Cell Signalling Technology, #30455) and β-actin (Proteintech, 66009-1-Ig) at 4 °C overnight. After incubation with the corresponding HRP-conjugated secondary antibodies (Proteintech, SA00001-1 and SA00001-2), the proteins were visualised using a chemiluminescence kit (Millipore, WBKLS0500) and a chemiluminescence detector. The expression of each protein was quantified using ImageJ software (v. 5.1) and normalised to the levels of β-actin.

### Non-targeted metabolomics analysis

Non-targeted metabolomics analysis was performed by Biomarker Technologies (Beijing, China). Briefly, 100 mg of piglet lung tissue samples were ground and added to an aqueous solution containing 80% methanol and 0.1% formic acid. Then, 20 µL of the liquid sample was extracted with 120 µL of pre-cooled 50% methanol. The samples were vortexed for 1 min, incubated at room temperature for 10 min, and stored overnight at −20 °C.

Following centrifugation at 4000 × *g* for 20 min, the supernatant was transferred to a 96-well plate and preserved at −80 °C prior to LC–MS analysis. Quality control samples were prepared by combining 10 µL from each extract. After sample preparation, the extracts underwent systematic analysis. Initially, the raw mass spectral data were converted into an interpretable format using ProteoWizard msConvert software [15], converting to mzXML. Compound annotation and preliminary identification were performed using CAMERA within the metaX software [16]. The putative identifiers were further annotated using databases such as HMDB and KEGG, which elucidate the metabolites’ physicochemical properties and biological functions. Differential metabolites were quantified and screened using metaX software.

### RNA sequencing (RNA-Seq)

The RNA**-**Seq was performed by Biomarker Technologies (Beijing, China). Briefly, piglet lungs were homogenised in liquid nitrogen, after which total RNA was extracted using TRIzol reagent. rRNA was removed using the Ribo-Zero rRNA Removal Kit (Illumina, MRZH116). Each specimen contributed 1 μg of the cDNA library. An Agilent 2100 Bioanalyzer was then used to estimate the size of the cDNA library. Finally, 150 bp paired-end reads were generated from the cDNA sequence via the Illumina HiSeq 4000 platform.

### Cell culture

The 3D4/21 PAMs were obtained from Guangzhou Runze Biotechnology Co. and seeded in RPMI 1640 medium (Gibco, 11875176). HEK293T cells were obtained from Hunan Fenghui Biotechnology Co. and seeded in Dulbecco’s Modified Eagle Medium (DMEM; Gibco, C11995500BT). Both media were supplemented with 10% foetal bovine serum (Gibco, 10099) and 1% penicillin–streptomycin (Solarbio, P1400). All cells were grown at 37 °C with 5% CO_2_. For the PAM in vitro cold-stimulation model, 1.5 mM menthol (MCE, HY-75161) was added. PAMs were infected with APP at an infection ratio of 10:1(bacterial cells to macrophages). Cells were stimulated for 3 h.

### Plasmid transfection

Cells were seeded in 6-cm cell culture dishes, and transfection was performed using Lipo8000^™^ transfection reagent (Beyotime Biotechnology, C0533FT) with 5 μg plasmid DNA and 8 μL of transfection reagent per dish. Cells were incubated for 48–72 h, with medium changes as required.

### Cell immunofluorescence

Tissue sections were immersed in 4% formaldehyde solution for 30 min at room temperature before staining. Then, the cells were permeabilised with Triton X-100 (BioFroxx, 1139) for 15 min and blocked for 1 h at room temperature. The sections were then immunostained with the primary antibody in a wet box overnight at room temperature, followed by incubation with the secondary antibody. Finally, 5 μL of antifade mounting medium with DAPI (Beyotime Biotechnology, P0131) was added dropwise to the slide, and the slides were sealed.

### Co-immunoprecipitation analysis

Transfected or non-transfected cells were lysed on ice for 30 min in NP-40 lysis buffer (Beyotime Biotechnology, P0013F), and centrifuged at 12 000 × *g* for 10 min. The pre-clarified supernatant was treated with the target antibody at 4 °C for 12 h. The antigen–antibody complex was then incubated for 1 h at room temperature with A/G magnetic beads (Thermo Fisher, 88803). Samples were washed three times with immunoprecipitation (IP) buffer, and the precipitated material was heated in SDS–PAGE sample buffer. Lysates were analysed by western blotting.

### Statistical analysis

All statistical parameters were calculated using GraphPad Prism 8.0 (GraphPad Software, USA). Values are expressed as the mean ± standard deviation (SD). The significance of differences among groups was determined by one-way or two-way analysis of variance (ANOVA). The asterisks indicate significant differences (*^/#^*P* < 0.05; **^/##^*P* < 0.01; ***^/###^*P* < 0.001; ****^/###^*P* < 0.0001; ns, not significant).

## Results

### Cold stimulation exacerbates the dysregulation of internal environmental homeostasis in piglets induced by APP infection

**Figure 1:**
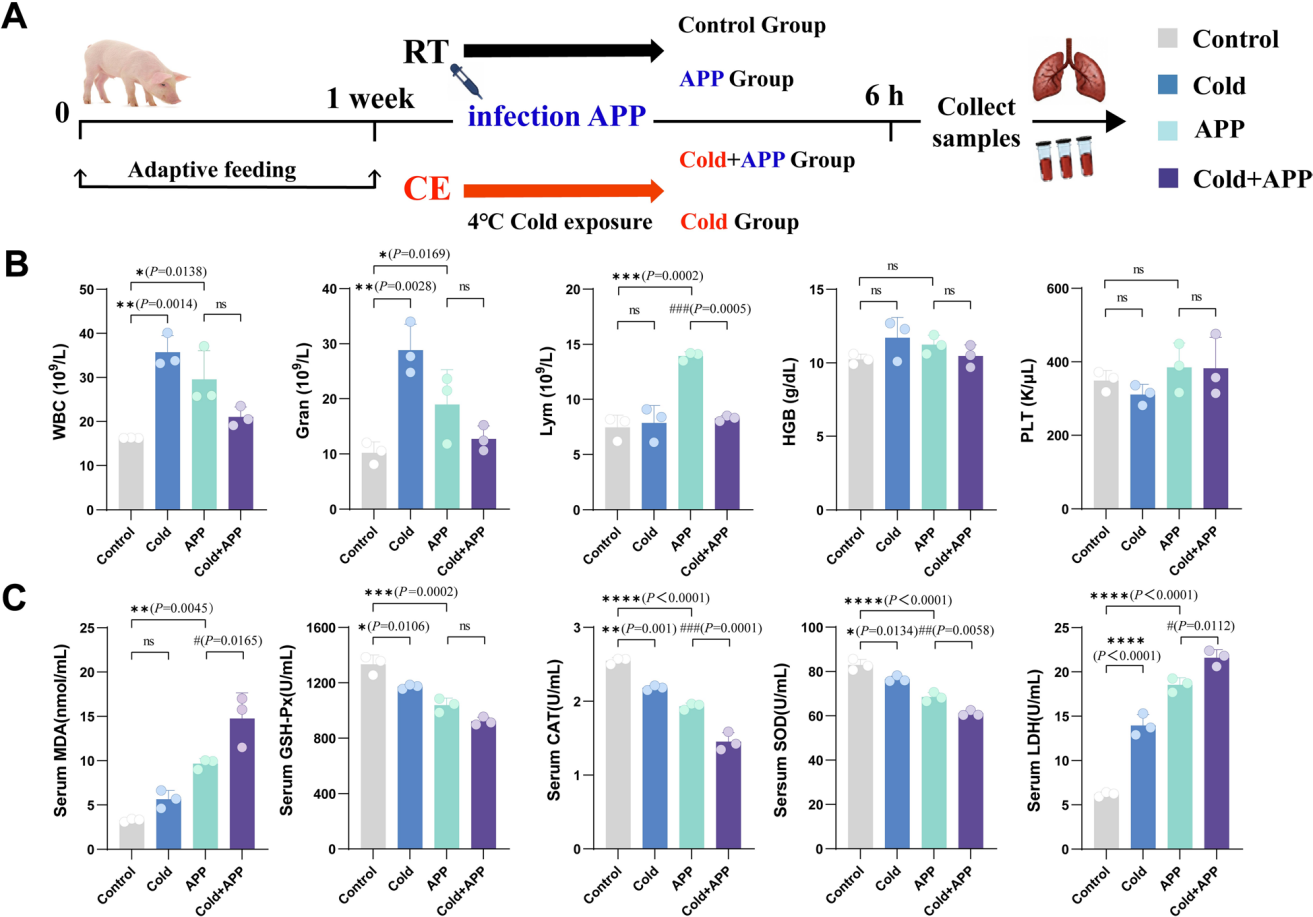
**Abnormal blood indices in piglets after APP infection under cold-stimulated conditions.** **A** Schematic illustration of the experimental design. **B** Routine examination for changes in the number of blood cells in the peripheral blood of piglets after 6 h of infection with APP under cold-stimulated conditions, in the order of white blood cell (WBC), neutrophilic granulocyte (Gran), lymphocyte (Lym), haemoglobin (HGB), platelets (PLT). **C** Expression levels of malondialdehyde (MDA), glutathione peroxidase (GSH-Px), catalase (CAT), superoxide dismutase (SOD), and lactate dehydrogenase (LDH) in the serum of piglets after APP infection under cold-stimulated conditions. The data are presented as the means ± SDs (*^/#^*P* < 0.05; **^/##^*P* < 0.01; ***^/###^*P* < 0.001; ****^/###^*P* < 0.0001; ns, not significant).

First, we systematically analysed the physiological state of piglets and changes in the lung internal environment using a cold-stimulation model in which piglets were infected with APP (2 × 10^9^ CFU/mL) via nasal drops (Figure 1A).

Cold-stressed animals exhibited behavioural responses, including huddling, crouching in corners, and shivering, along with a drop in skin temperature and changes in skin colouration. We then quantified changes in peripheral blood indicators. Cold stimulation significantly increased white blood cell (WBC) and neutrophil (Gran) counts in the blood, whereas the APP-infected group showed a decreasing trend in WBC and Gran counts compared with the Cold group. Notably, piglets in the Cold + APP group exhibited lower WBC, Gran, and lymphocyte (Lym) counts than those in the APP group (Figure 1B), indicating that cold stimulation increases the susceptibility of piglets to APP infection. Serum biochemical analyses in piglets showed that both cold stimulation and APP infection increased MDA and LDH levels while decreasing GSH-Px, CAT, and SOD activities. Moreover, compared with the APP group, the Cold + APP group exhibited a further significant upregulation of MDA and LDH levels, and a marked downregulation of CAT and SOD activities (Figure 1C). These findings indicate that APP infection under cold stimulation leads to abnormal blood metabolic indices in piglets and that cold stimulation further disrupts in vivo homeostasis in APP-infected piglets.

### Cold stimulation exacerbates lung injury induced by APP infection in piglets

**Figure 2:**
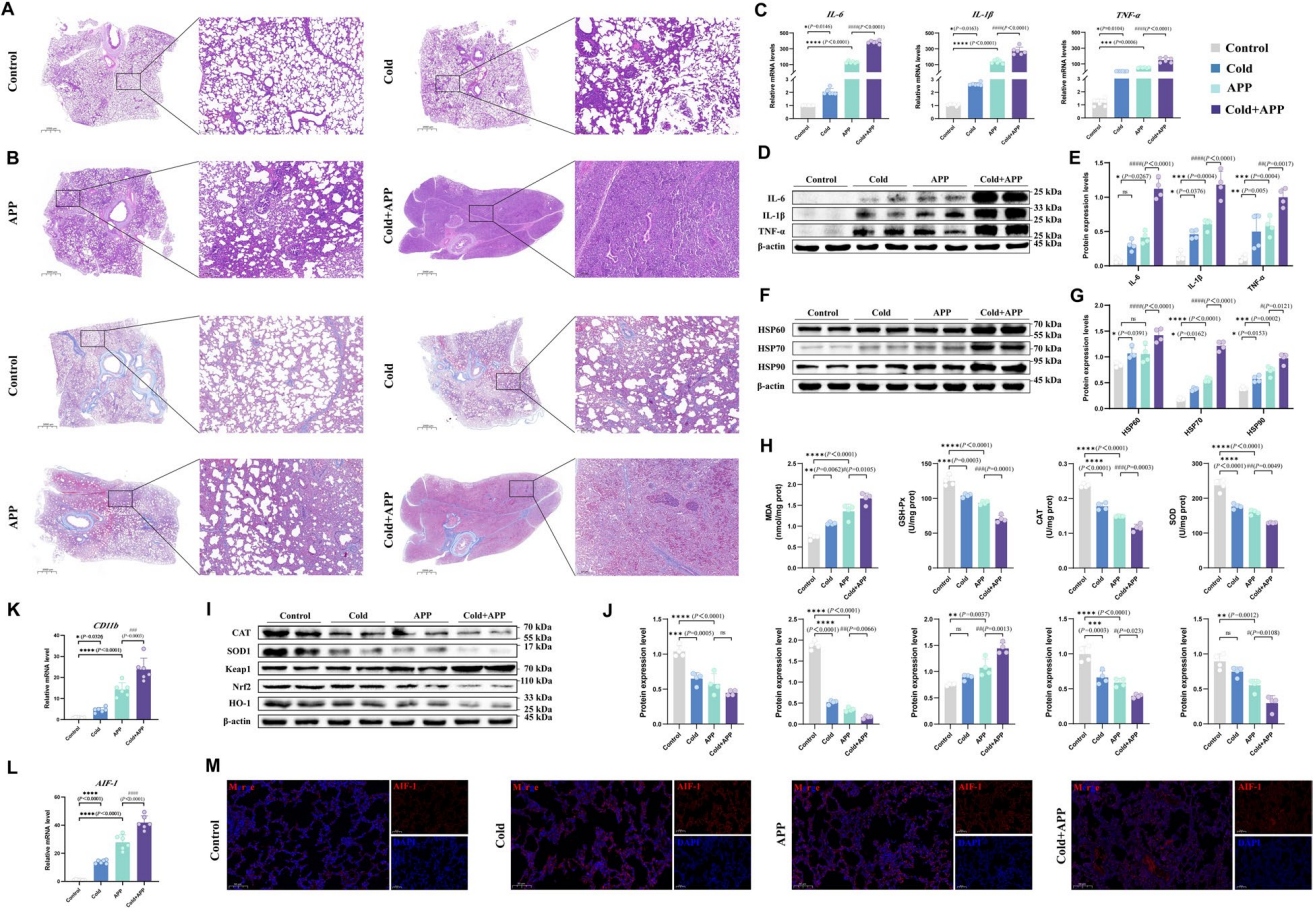
**Cold stimulation exacerbates lung injury induced by APP infection and promotes macrophage activation.** **A** Representative images of HE staining of the lungs of APP-infected piglets under cold stimulation conditions. Scale bar = 200 μm. **B** Representative images of Masson staining of the lungs of APP-infected piglets under cold stimulation conditions. Scale bar = 200 μm. **C** qRT-PCR detection of inflammatory factors (*IL-6*, *IL-1β*, and *TNF-α*) mRNA expression in piglet lungs. **D** and **E** Western blot assays of inflammatory factors (IL-6, IL-1β and TNF-α) in piglet lungs. **F** and **G** Western blot assays of heat shock proteins (HSP60, HSP70 and HSP90) in piglet lungs. **H** Biochemical kits for the detection of oxidative stress indicators (MDA, GSH-Px, CAT and SOD) in piglet lungs. **I** and **J** Western blot assays of antioxidant proteins (CAT, SOD1, Keap1, Nrf2 and HO-1) in piglet lungs. **K** and **L** qRT-PCR detection of the mRNA expression of activation markers of pulmonary macrophages (*CD11b* and *AIF-1*) in piglet lungs. **M** Representative images of immunofluorescence staining for AIF-1 (red) and DAPI (blue) in piglet lungs. Scale bar = 50 μm. The data are presented as the means ± SDs (*^/#^*P* < 0.05; **^/##^*P* < 0.01; ***^/###^*P* < 0.001; ****^/###^*P* < 0.0001; ns, not significant).

Having established that cold stimulation can mediate alveolar macrophage (AM) activation and induce lung inflammation, we further investigated how APP infection affects the lung immune response of piglets under cold-stimulation conditions [17]. Pronounced lesions were observed in the lungs of piglets in the Cold + APP group, characterised by inflammatory cell infiltration and fibroblast proliferation, which coalesced to form solid foci (Figure 2A, B). Concentrations of the pro-inflammatory cytokines IL-6, IL-1β, and TNF-α were released in large quantities in the lung tissue of piglets in the Cold + APP group (Figure 2C–E). As heat-shock proteins are key indicators of stress, we assessed their expression levels in the lung tissues of piglets in the Cold + APP group and found significant upregulation compared with those in the APP group (Figure 2F, G). This suggests that cold stimulation further disrupts lung homeostasis in piglets following APP infection. Indicators of oxidative stress in the lungs were then assessed. The results showed that lipid peroxidation was markedly enhanced under cold-stimulation conditions following APP infection, whereas antioxidant enzymes (GSH-Px, CAT, SOD) were significantly depleted (Figure 2H). This suggests that cold stimulation exacerbates APP infection-induced oxidative stress damage and reduces antioxidant capacity in piglet lungs. Correspondingly, the protein expression levels of antioxidant-related enzymes, including CAT, SOD1, Keap1, Nrf2, and HO-1, further support the conclusion that piglets are in a state of oxidative stress (Figure 2I, J).

Macrophages are essential for maintaining the dynamic balance of the immune system and play a key role in the initiation, resolution, and tissue repair of lung inflammation [18]. In piglets exposed to cold stimulation, the mRNA transcript levels of the macrophage activation markers *CD11b* and *AIF-1* were significantly increased in the lungs after APP infection under cold-stimulation conditions (Figure 2K, L). The distribution of AIF-1 in lung tissue was further detected by immunofluorescence (Figure 2M). Together, these findings indicate that cold stimulation acts as a conditioning factor that promotes enhanced macrophage activation during APP-induced lung inflammation.

### Immune cells in the lungs of APP-infected piglets under cold stimulation can interfere with immune function by altering metabolic pathways

**Figure 3:**
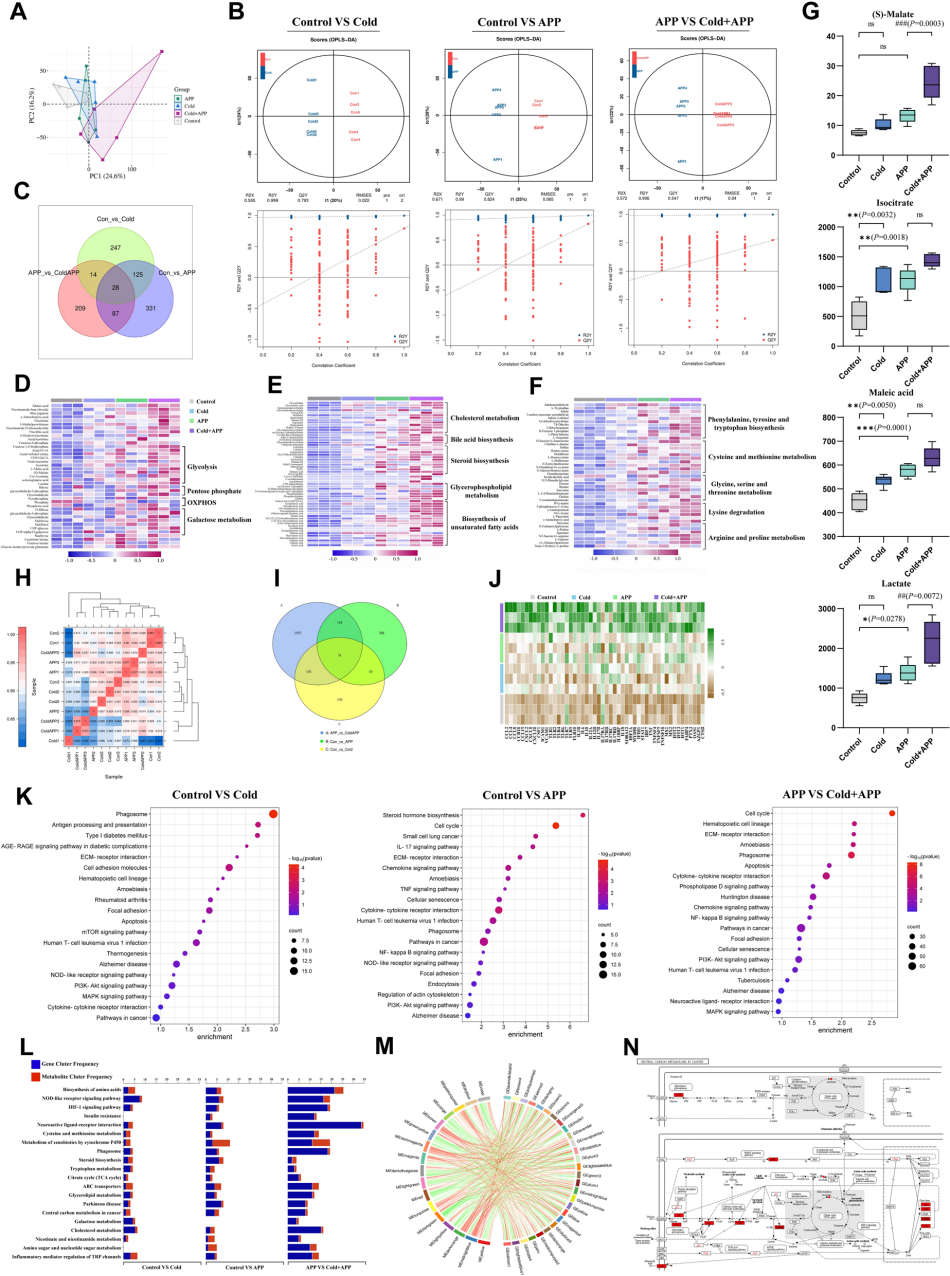
**Intervention in immune function through metabolic reprogramming of cells in the lungs of APP-infected piglets under cold stimulation.** **A** Metabolite principal component analysis score plot. **B** Orthogonal partial least squares discriminant analysis of metabolomic data, score plots (up) and replacement charts (down). **C** Venn diagrams of differential metabolites for different combinations. **D–F** Heatmap of clustering of differential metabolites involved in glucose, lipid, and amino acid metabolism. Horizontal coordinates indicate the subgroups in which the samples are located, and vertical coordinates represent the differential metabolites. **G** Metabolomics-based box plot analysis of glycolytic products. From top to bottom, (S)-malate, isocitrate, maleic acid, lactate. **H** Heatmap of expression correlation between two-by-two samples in transcriptomics. **I** Venn diagrams of differential genes for different comparison combinations. **J** Heatmap of differentially expressed genes involved in immune metabolism. **K** Bubble diagram of the top 20 KEGG pathways for differentially expressed genes. **L** KEGG co-enrichment analysis of gene and metabolite expression differences. The horizontal coordinates are the number of differential gene clusters and differential metabolite clusters, with blue representing genes and red representing metabolites, and the vertical coordinates are KEGG pathways. **M** Correlation chord plots of metabolite module/gene module data for the top 30 frequencies (ordered by the absolute value of correlation coefficients from largest to smallest). Metabolites are shown in the left half circle of the chord diagram, and genes in the right half circle. Each string indicates that the metabolite module is significantly correlated with the gene module, with the red string representing a positive correlation and the green string representing a negative correlation. **N** Mesh diagram of the central carbon metabolic pathway in cancer. Boxes indicate genes, circles indicate metabolites, and red indicates upregulated genes/metabolites.

A close interplay exists between metabolism and immune function, and immune dysfunction is a contributing factor in the development of metabolic disorders [19]. We therefore hypothesised that APP infection under hypothermic conditions may exacerbate lung injury by altering the metabolic flux in the lungs. Untargeted metabolomics profiling by LC-MS/MS was performed to identify and analyse differential metabolites in the lungs of piglets from each group (Figure 3A–C). Additionally, we performed a heatmap analysis of sugar, lipid, and amino acid metabolites, revealing that metabolic fluxes were altered in the lungs of piglets following APP infection under cold-stimulation conditions. Glucose-to-lactate metabolism (glycolysis) and the pentose phosphate pathway were enhanced, while oxidative phosphorylation was inhibited. This finding is consistent with the ‘Warburg effect’ induced by immune cell activation. Lipid-metabolism analysis revealed increased cholesterol metabolism and fatty acid biosynthesis. In amino acid metabolism, related pathways such as glycine, serine, and threonine metabolism, as well as cysteine and methionine metabolism, were upregulated (Figure 3D–F). Additionally, we analysed the metabolite expression of the tricarboxylic acid cycle to further validate the above results (Figure 3G).

Differentially expressed genes (DEGs) were identified by RNA-Seq in the lungs of piglets from each group, and immune-related genes were visualised (Figure 3H–J). The results showed that genes associated with immune processes, including those encoding cytokines, chemokines, Toll-like receptors, and interleukins, were significantly upregulated following APP infection under cold-stimulation conditions. Enrichment analysis of KEGG biological pathways showed that the DEGs following cold stimulation were mainly enriched in pathways related to cell-adhesion molecules and antigen processing and presentation. DEGs induced by APP infection were enriched predominantly in pathways associated with the cell cycle and cytokine–cytokine receptor interaction. Compared with the APP-infected group, the DEGs following APP infection under cold-stimulation were enriched in biological pathways such as the cell cycle, the haematopoietic cell lineage, and cytokine–cytokine receptor interactions (Figure 3K). Most of these pathways are associated with immune processes and inflammatory responses. Combined with our previous findings of extensive pro-inflammatory cytokine secretion in the lungs, we suggest that a ‘cytokine storm’ likely occurred as a result of APP infection.

To further explore, we conducted co-enrichment analysis by constructing differential gene-expression and metabolite-expression profiles. The number of differential gene clusters and differential metabolite clusters involved in the top 20 KEGG pathways containing the highest number of co-participants was visualised for intergroup comparisons (Figure 3L). The results showed that the number of differential gene clusters and differential metabolite clusters in the APP versus Cold + APP co-enrichment increased significantly compared with the Control versus Cold and Control versus APP co-enrichment. Consequently, we focused the subsequent analyses on the DEGs and metabolites in the APP versus Cold + APP co-enrichment. These differential genes and metabolites were divided into distinct modules, and the correlation results for the metabolite and gene modules with the top 30 frequencies were used to construct correlation chord plots. A strong positive correlation was observed between the metabolite module and gene modules (Figure 3M).

KEGG network integration of differential genes and metabolites revealed that pathways associated with glycolysis and fatty acid biosynthesis were upregulated within central carbon metabolism in cancer. This metabolic pathway is fundamental for maintaining normal cellular growth in organisms, and suggests a link to the "Warburg effect" (Figure 3N). These findings support our hypothesis that metabolic reprogramming of immune cells in the lungs occurs in response to energy mobilisation following APP infection.

### SIRT2 modulates the level of NF-κB p65 acetylation involved in the regulation of immune metabolism in piglet lungs

Metabolic pathways influence epigenetic gene regulation. The APP-S1 serotype I strain used in this study is dependent on NAD + for growth. Sirtuins, a class of deacetylases that regulate protein function through NAD-dependent post-translational modifications, are highly sensitive to changes in the intracellular metabolic microenvironment [20].

**Figure 4:**
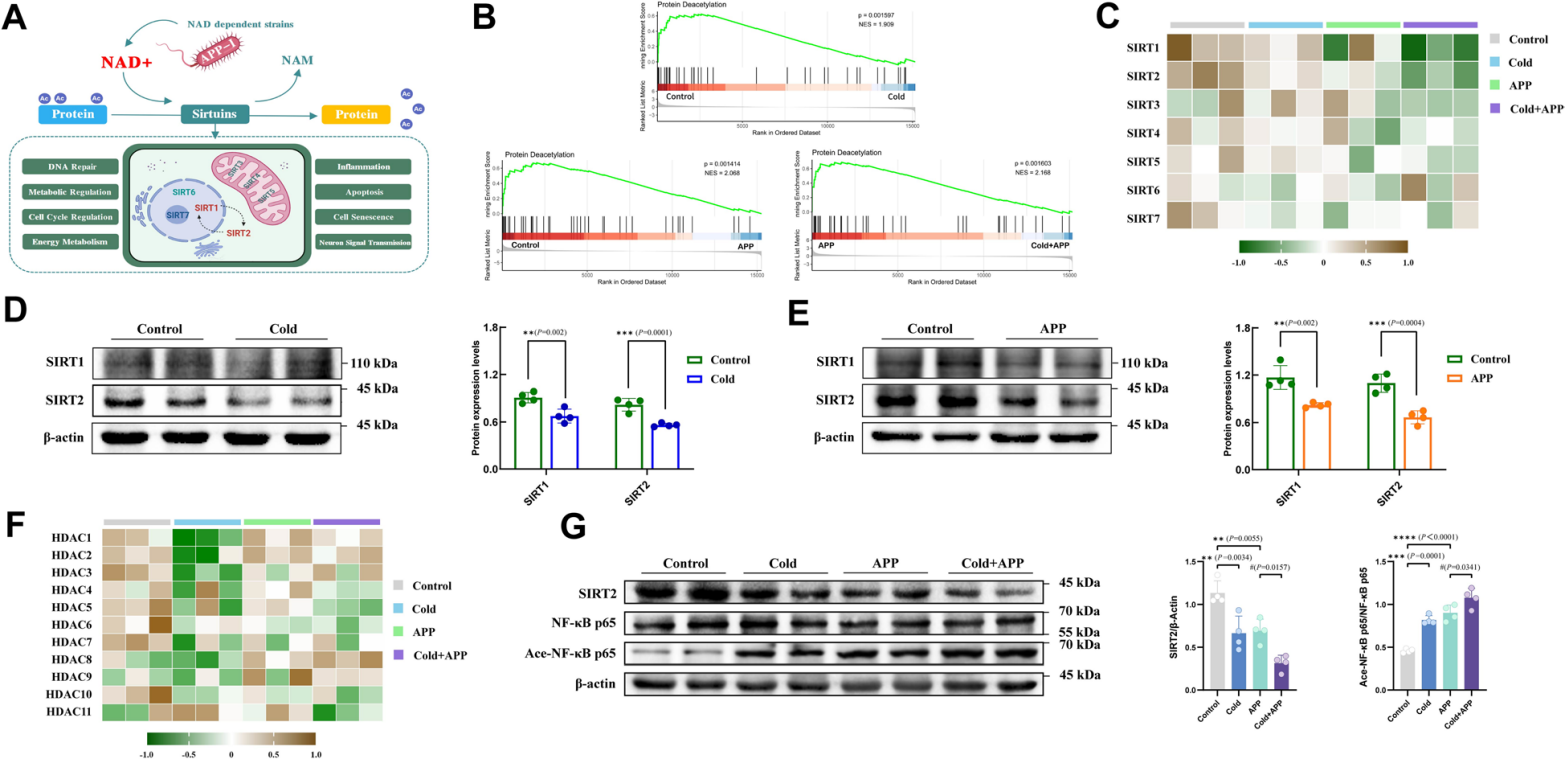
**SIRT2 is specifically involved in lung immune metabolism in APP-infected piglets under cold-stimulated conditions.** **A** Schematic illustration of the relationship between APP and sirtuins. **B** Gene set enrichment analysis map of the protein deacetylation pathway. **C** Transcriptomics-based heatmap of SIRT1–SIRT7 gene expression. **D** Western blot assays for SIRT1 and SIRT2 in piglet lung tissue under cold stimulation. **E** Western blot assays for SIRT1 and SIRT2 in piglet lung tissue after APP infection. **F** Transcriptomics-based heatmap of HDAC1–11 gene expression. **G** Western blot assays for SIRT2 and Ace-NF-κB p65 expression levels in piglet lungs. The data are presented as the means ± SDs (*^/#^*P* < 0.05; **^/##^*P* < 0.01; ***^/###^*P* < 0.001; ****^/###^*P* < 0.0001; ns, not significant).

On this basis, we established a connection between APP and sirtuins (Figure 4A). Gene set enrichment analysis revealed that APP infection under cold-stimulated conditions significantly downregulated protein-deacetylation processes, suggesting that sirtuins can specifically participate in the regulation of lung immune function (Figure 4B). Subsequently, RNA-Seq-based heatmap analysis was performed on the seven members of the sirtuin family (SIRT1-SIRT7). Among these, the expression of SIRT1 and SIRT2 was significantly downregulated following cold-stimulated APP infection (Figure 4C). As SIRT1 and SIRT2 can shuttle between the nucleoplasm and cytoplasm, we further examined their expression under both cold stimulation and APP infection conditions. The results showed that the protein expression levels of SIRT1 and SIRT2 were significantly downregulated under both conditions, with SIRT2 exhibiting more pronounced downregulation (Figure 4D, E). This outcome may be attributed to the cytoplasmic localisation of SIRT2, which enables its direct involvement in metabolic processes. These findings identify SIRT2 as a key regulator of the pulmonary immune response during APP infection under cold-stimulated conditions.

In addition to sirtuins, the enzymes involved in deacetylation include histone deacetylases (HDACs), which are typically zinc-dependent metalloenzymes (HDAC1–11). Among these, HDAC1, HDAC2, and HDAC3 are the core isoforms. These isoforms exist as multiprotein complexes in the nucleus, where they deacetylate histones and transcriptional regulators, and are ubiquitous across life forms. Our heatmap analysis of HDAC1–11 revealed that the expression of HDAC1, HDAC2, and HDAC3 was significantly downregulated under cold stimulation conditions. However, their expression did not differ between the APP and Control groups. The expression levels of HDAC4–11 were not statistically different between the experimental groups (Figure 4F). Based on these findings, we excluded the possibility that HDACs regulate the lung immune response following APP infection.

NF-κB p65 (Lys310) is a key transcriptional regulator with broad biological effects, participating not only in regulating inflammation but also in fine-tuning molecular modulation [21]. Consistent with this, the NF-κB signalling pathway was enriched following APP infection, as well as APP infection under cold-stimulation conditions (Figure 3G). We therefore examined whether SIRT2 modulates NF-κB p65 activity through deacetylation. Using an antibody that specifically detects acetylation of NF-κB at Lys310, we found that SIRT2 inhibited the deacetylation of NF-κB p65 following APP infection under cold-stimulation conditions, resulting in increased acetylation levels (Figure 4G).

### Inhibition of SIRT2 amplifies the immune response to PAMs following APP infection

PAMs are the most abundant immune cell type in healthy lungs and can manipulate changes in cellular metabolism to determine the immune response [22]. SIRT2, a key metabolic sensor, has been shown to play a crucial role in regulating macrophage fate across diverse metabolic microenvironments [23]. Its activity in response to cold stimulation is a key factor influencing the immune response of PAM following APP infection.

**Figure 5:**
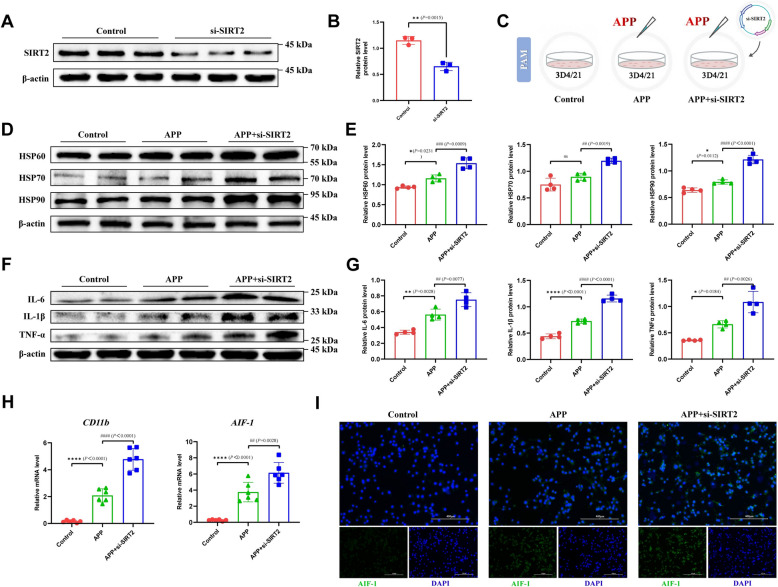
**Effect of SIRT2 on the immune status of PAMs during APP infection. A** and** B** Western blot assays for SIRT2 expression level in PAMs after transfection with si-SIRT2. **C** Schematic illustration of APP-infected PAMs. **D** and **E** Western blot assays for heat shock protein (HSP60, HSP70, and HSP90) in PAMs after APP infection. **F** and **G** Western blot assays for inflammatory factors (IL-6, IL-1β and TNF-α) in PAMs after APP infection. **H** qRT-PCR detection of the mRNA expression of activation markers of pulmonary macrophages (*CD11b* and *AIF-1*) in PAMs after APP infection. **I** Representative images of immunofluorescence staining for AIF1 (red) and DAPI (blue) within PAMs after APP infection. Scale bar = 400 μm. The data are presented as the means ± SDs (*^/#^*P* < 0.05; **^/##^*P* < 0.01; ***^/###^*P* < 0.001; ****^/###^*P* < 0.0001; ns, not significant).

To determine the effect of SIRT2 on PAM function after APP infection, we designed siRNA targeting SIRT2 and transfected it into PAMs, thereby knocking down SIRT2 expression (Figure 5A. B). Following knockdown, PAMs were infected with APP (Figure 5C), and the expression of heat-shock proteins and inflammation-associated proteins in PAMs was assessed. The results showed that APP infection significantly upregulated the expression of these proteins, and the presence of siSIRT2 further increased their expression levels after APP infection (Figure 5D–G). These findings indicate that knockdown of SIRT2 exacerbates the dysregulation of PAM homeostasis induced by APP infection and amplifies the immune response effects.

Based on our in vivo finding that cold stimulation enhances APP infection-induced macrophage activation in piglet lungs, we further investigated whether SIRT2 knockdown similarly enhances the activation of APP-infected PAMs at the cellular level. qRT-PCR analysis showed that APP infection following SIRT2 knockdown significantly increased the mRNA transcript levels of *AIF-1* and *CD11b* compared with the APP-infected group (Figure 5H). Immunofluorescence analysis of AIF-1 further supported this result: its fluorescence intensity was substantially increased following reinfection with APP under SIRT2 knockdown conditions (Figure 5I). Together, these findings indicate that inhibition of SIRT2 promotes PAM activation in response to APP infection.

### SIRT2 interacts with NF-κB p65 to mediate acetylation modifications

**Figure 6:**
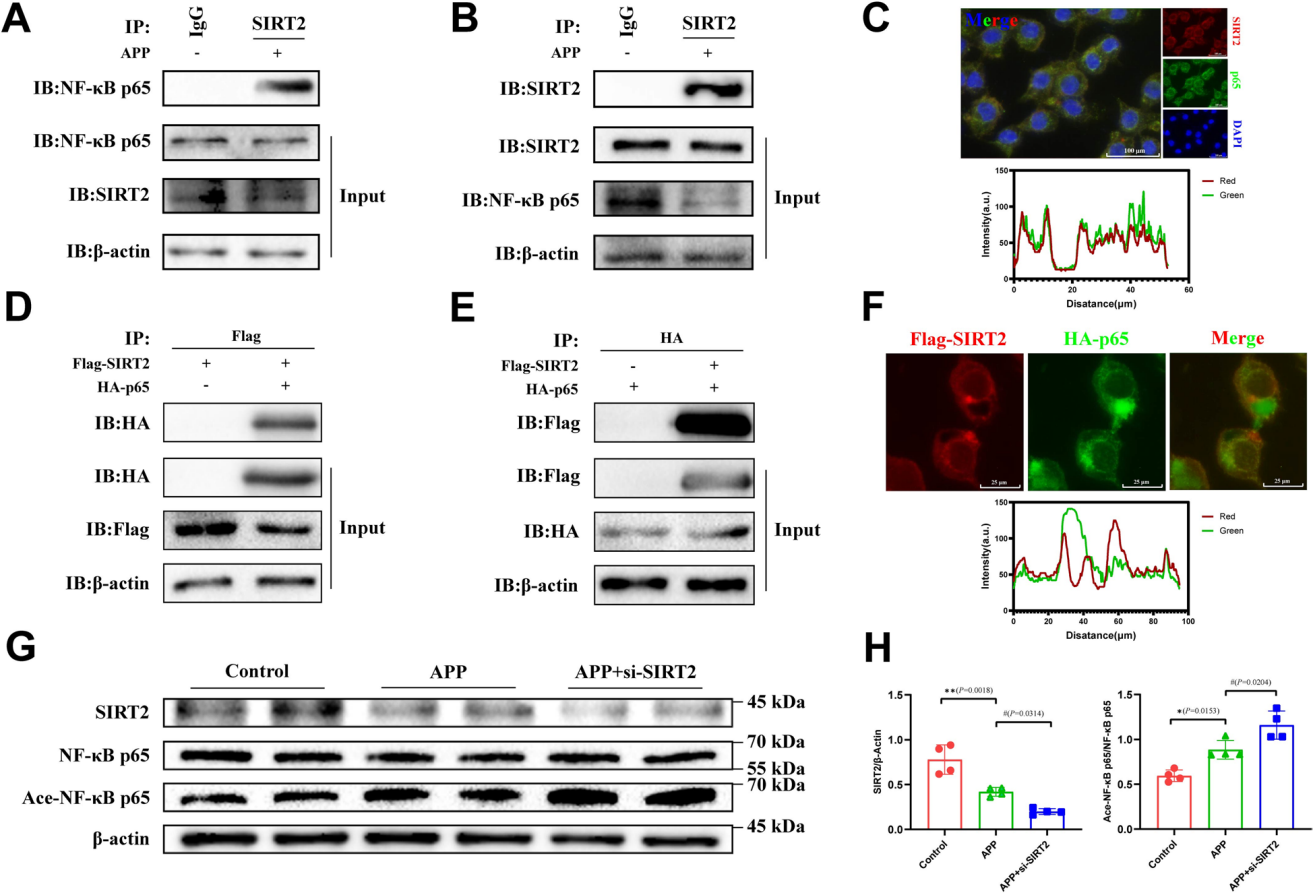
**Validation of the interaction between SIRT2 and NF-κB p65.** **A** and **B** Immunoprecipitation results of SIRT2 and NF-κB p65 in PAMs following APP infection. **C** Immunofluorescence detection of SIRT2 (red) and NF-κB p65 (green) within PAMs to validate the interplay between SIRT2 and NF-κB p65. Scale bar = 100 μm. **D** and **E** HEK293T cells were transfected with plasmids expressing Flag-SIRT2 or HA-NF-κB p65, and samples were collected after 48 h. The exogenous interaction of SIRT2 with NF-κB p65 was detected by a pulldown assay. **F** Fluorescence of each fusion protein in HEK293T cells was observed by fluorescence microscopy. Colocalisation of Flag-SIRT2 and HA-NF-κB p65 in the merged image is shown in yellow. Scale bar = 25 μm. **G** and **H** Western blot assays of the expression levels of SIRT2 and Ace-NF-κB p65 in SIRT2-knockdown PAMs after APP infection. The data are presented as the means ± SDs (*^/#^*P* < 0.05; **^/##^*P* < 0.01).

Based on our in vivo findings, we next examined whether SIRT2 affects the immune response of PAMs during APP infection by modulating the activity of the NF-κB p65 subunit. First, we investigated the interaction patterns between SIRT2 and NF-κB p65. PAMs were infected with APP, and SIRT2 and NF-κB p65 were used as bait proteins for immunoprecipitation to detect their protein–protein interactions (Figure 6A, B). The colocalisation of SIRT2 with NF-κB p65 was further confirmed using fluorescence confocal microscopy (Figure 6C).

Next, exogenous Flag-SIRT2 and HA-NF-κB p65 were transfected into HEK-293 T cells and cell lysates for pulldown assays were collected after 24 h of culture. The results demonstrated that exogenous Flag-SIRT2 and HA-NF-κB p65 could bind to each other (Figure 6D, E). Fluorescence colocalisation analysis further confirmed that the two proteins colocalised within cells (Figure 6F). We then assessed whether SIRT2 binding to NF-κB p65 within PAMs alters its acetylation modification level following APP infection. Consistent with our in vivo results, the protein expression of acetylated NF-κB p65 (Ace-NF-κB p65) was significantly upregulated in APP-infected PAMs, concomitant with the downregulation of SIRT2 (Figure 6G, H). These findings suggest that SIRT2 mediates the acetylation modification of NF-κB p65 after APP infection and suppresses its deacetylation.

### Retrospective validation of SIRT2 regulation of lung immune strength after APP infection

**Figure 7:**
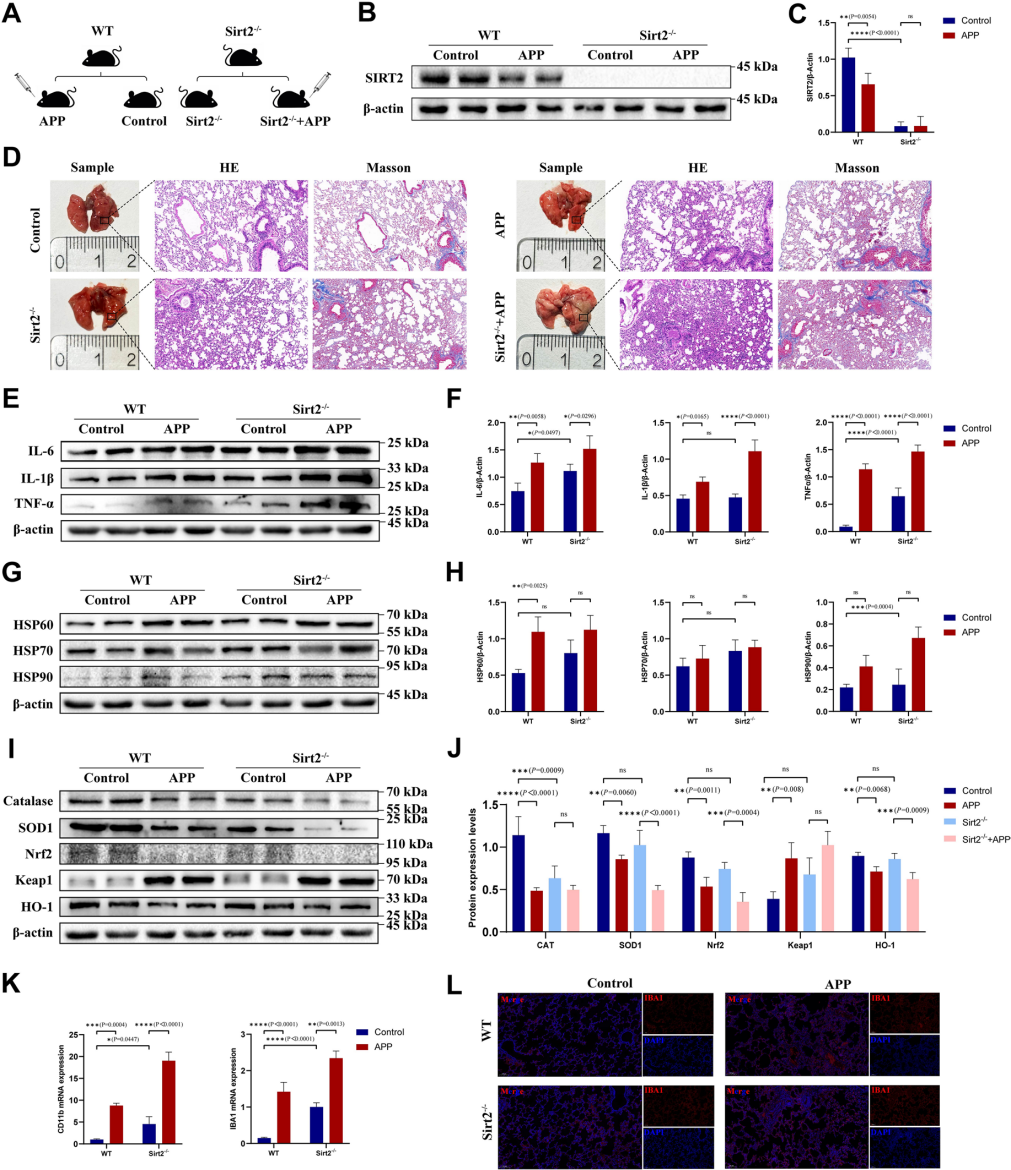
**APP-infected mice exhibit more severe lung injury with concomitant macrophage activation after *Sirt2*** **knockout**. **A** Schematic illustration of the experimental design. **B** and **C** Western blot assays for SIRT2 in the lungs of wild-type mice (WT) and SIRT2-knockout mice (*Sirt2*^*−*/*−*^) after infection with APP and examination of the knockout efficiency. **D** Representative images of fresh lung tissue, HE staining, and Masson staining of mice after infection with APP in WT and *Sirt2*^*−*/*−*^ mice, respectively. **E** and **F** Western blot assays for inflammatory factors (IL-6, IL-1β and TNF-α) in the lungs of WT and *Sirt2*^*−*/*−*^ mice after infection with APP. **G** and **H** Western blot assays for inflammatory factors (HSP60, HSP70 and HSP90) in the lungs of WT and *Sirt2*^*−*/*−*^ mice after infection with APP. **I** and **J** Western blot assays for antioxidant proteins (CAT, SOD1, Keap1, Nrf2, and HO-1) in the lungs of WT and *Sirt2*^*−*/*−*^ mice after infection with APP. **K** qRT-PCR detection of the mRNA expression of activation markers of pulmonary macrophages (*CD11b* and *IBA1*) in the lungs of WT and *Sirt2*^*−*/*−*^ mice after infection with APP. **L** Representative images of immunofluorescence staining for IBA1 (red) and DAPI (blue) in the lungs of WT and *Sirt2*^*−*/*−*^ mice after infection with APP. Scale bar = 50 μm. The data are presented as the means ± SDs (*P < 0.05; **P < 0.01; ***P < 0.001; ****P < 0.0001).

Having shown that SIRT2 mediates the PAM immune response to APP infection by regulating the acetylation modification of NF-κB p65, we next evaluated the role of SIRT2 in modulating the lung immune response after APP infection, using infected wild-type (WT) and *Sirt2* knockout mice (*Sirt2*^*−*/*−*^) with APP (1 × 10^7^ CFU) (Figure 7A). In WT mice, SIRT2 protein expression was significantly reduced in the lungs following APP infection, whereas no SIRT2 protein expression was detected in *Sirt2*^*−*/*−*^ mice (Figure 7B, C).

The sampling images revealed clear lesions in the lungs of APP-infected *Sirt2*^*−*/*−*^ mice, and pathological analysis indicated that *Sirt2* knockout exacerbates lung injury induced by APP infection (Figure 7D, E). Pro-inflammatory cytokine expression assays demonstrated that *Sirt2* knockdown enhances the secretion of inflammatory factors in the lung tissues of APP-infected mice and disrupts the homeostasis of the pulmonary internal environment (Figure 7E–H). *Sirt2* knockout also impaired the activation of antioxidant enzymes in the lungs of APP-infected mice, leading to increased oxidative stress (Figure 7I, J).

Finally, analysis of macrophage activation markers found that *Sirt2* knockdown enhanced APP infection-induced macrophage activation compared with WT mice (Figure 7K, L). Collectively, these findings demonstrate that SIRT2 plays an important role in regulating the strength of the immune response in the lungs following APP infection.

## Discussion

Low environmental temperatures are a major stressor for livestock and poultry during autumn and winter in colder regions, contributing substantially to the rise in various respiratory diseases in young animals exposed to cold stress [24]. APP, a principal pathogen implicated in PRDC, remains prevalent globally and continues to impact the swine industry severely. Accordingly, strategies to mitigate APP-induced inflammatory damage and strengthen the host’s immune response remain of considerable importance. Cold-based stimulation itself can cause lung injury by triggering oxidative stress, promoting inflammation in lung tissue, and disrupting the barrier function of the host’s natural immune system [25]. Here, we demonstrate for the first time that cold stimulation acts as a conditioning factor that exacerbates APP-induced lung damage in piglets. A dynamic equilibrium exists between the circulating and marginal pools through which WBCs develop. Notably, our findings revealed a significant increase in the number of WBCs following cold stimulation, suggesting that physiological factors such as cold stress lead to a sharp rise in catecholamine levels, which mobilise leukocytes from the marginal pool into circulation.

In contrast, WBC and Gran counts decreased in organisms subjected to APP infection, either alone or in combination with cold stimulation, than in those subjected to cold stimulation alone. This may reflect immune activation and the adhesion of some leukocytes to endothelial cells, thus increasing the retention of the marginal pool of leukocytes and corresponding decreases in the measured circulating pool. Such redistribution amplifies the inflammatory response, thereby combating the invasion of exogenous pathogens. These findings also explain the significant increase in susceptibility to APP low-temperature conditions, which further exacerbates the secretion of inflammatory factors following APP infection. Ultimately, this leads to a ‘cytokine storm’ and exacerbates pathological changes in the lungs of piglets.

A cytokine storm is an excessive immune response triggered by a positive feedback loop between cytokines and immune cells [26]. During this process, immune-associated cells such as lymphocytes and macrophages undergo continuous activation and expansion, resulting in excessive secretion of large amounts of cytokines. This results in systemic inflammatory response syndrome, multiorgan failure, and secondary symptoms such as acute respiratory distress in the host [27]. It has been shown that among the various pathogens capable of inducing PRDC, including APP, PRRSV, and *Mycoplasma pneumoniae*, all exhibit seasonal epidemiological characteristics, with higher prevalence during winter. These infections are often accompanied by severe PAM infiltration and the cytokine-storm-like responses [28]. Novel coronavirus pneumonia (COVID-19) shows a similar pattern, exhibiting higher incidence and infectiousness during the winter months, characterised by the hallmark aggregated activation of AMs following infection [29]. Furthermore, studies have shown that cold stimulus-induced lung injury can interfere with respiratory defences by damaging cilia and attenuating macrophage activity [30]. In our study, we found that cold stimulation promotes the activation of lung macrophages following APP infection, suggesting that disruption of lung homeostasis rapidly drives macrophages from a resting state to an activated state, strengthening non-specific host defences and initiating repair mechanisms.

Immune cell activation, differentiation, and function depend on an energy supply and metabolic transformation. By integrating untargeted metabolomics with transcriptomics, we constructed differential metabolite-gene expression profiles in the lung. The metabolomics results suggest that the organism may alter metabolic flux following APP infection by regulating metabolic switches and implementing modifications. This metabolic modification is consistent with the ‘Warburg effect’ induced by immune cell activation. Metabolism serves as a dominant force in immune regulation. RNA-Seq results revealed that metabolic pathways involved in the proliferation, differentiation, and effector functions of immune cells were enhanced following cold-stimulated APP infection. This suggests that macrophage dysfunction driven by metabolic abnormalities is a key factor affecting host immunity. APP infection also induced a cytokine-driven synergistic cascade that amplified the inflammatory response, leading to a cytokine storm that impairs the body's immune function [31]. We also observed enrichment of cytokine–cytokine receptor interactions in KEGG enrichment analyses, further demonstrating that cold-stimulated APP infection involves a combination of multiple cytokines. Therefore, we propose that the organism regulates AMs’ immune function through cellular metabolic reprogramming following cold-stimulated APP infection. Given that the APP strain used in this study is serotype I and dependent on NAD + for growth, and that sirtuins are NAD-dependent deacetylases, we identified SIRT2 as a key regulator of APP infection.

SIRT2 is known to modulate macrophage immune phenotype and function across various metabolic microenvironments [32, 33]. Therefore, SIRT2 activity within PAMs under cold stimulation may be a critical factor influencing the PAM immune response following APP infection. In our study, SIRT2 expression was downregulated following APP infection, with a more pronounced reduction under cold stimulation conditions. To investigate whether SIRT2 contributes to the regulation of PAM immune status following APP infection, we performed SIRT2 knockdown in vitro and simultaneously infected them with APP. The results showed that inhibition of SIRT2 upregulated the levels of post-infection stress and inflammatory factor expression induced by APP, leading to an increase in activated PAMs. As macrophage activation is closely related to the maintenance of tissue homeostasis, these findings indicate that inhibition of SIRT2 promotes persistent pro-inflammatory stimuli induced by APP infection and amplifies innate or acquired immune responses.

A bidirectional closed-loop regulatory mechanism may operate between metabolic enzyme acetylation modifications and metabolic pathways. A growing body of research suggests that SIRT2 can deacetylate a wide range of substrates and plays a key role in multiple pathological processes, including apoptosis, autophagy, and inflammatory immune responses [34]. In terms of inflammatory processes, SIRT2 has been shown to deacetylate NF-κB p65, thereby attenuating the inflammatory response in a brain injury model, renal tubular inflammation, and ischemia-reperfusion-induced hepatocellular inflammation [35–37]. NF-κB p65 is broadly present in all tissues of the body, and the NF-κB signalling pathway is activated in response to injury or infection. In addition, LPS, a virulence factor of APP, can induce pro-inflammatory cytokine production by PAMs through a TLR4/NF-κB-mediated pathway [38].

Consistent with this, KEGG enrichment analysis of DEGs in our study revealed enrichment of the NF-κB signalling pathway. We therefore propose that SIRT2 may utilise its deacetylation activity to modify the downstream target NF-κB p65, thereby interfering with the immune strength and sensitivity of PAMs following APP infection in cold-stimulated piglets. Supporting this hypothesis, we confirmed a direct interaction between SIRT2 and NF-κB p65. Both in vitro and in vivo, we observed that the deacetylation activity of SIRT2 was inhibited after APP infection, while the acetylation level of NF-κB p65 was upregulated. Furthermore, the interaction between SIRT2 and NF-κB p65 was mediated by the deacetylation function of SIRT2. Future work will include constructing SIRT2-catalysed mutants and NF-κB p65 structural domain-deletion mutants to confirm that elevated NF-κB p65 acetylation is dependent on the attenuation of SIRT2 deacetylation.

In conclusion, this study identifies, for the first time, the mechanism by which SIRT2 acetylation is altered under low-temperature conditions and its regulatory role in the immunoreactivity of PAMs.

**Figure 8:**
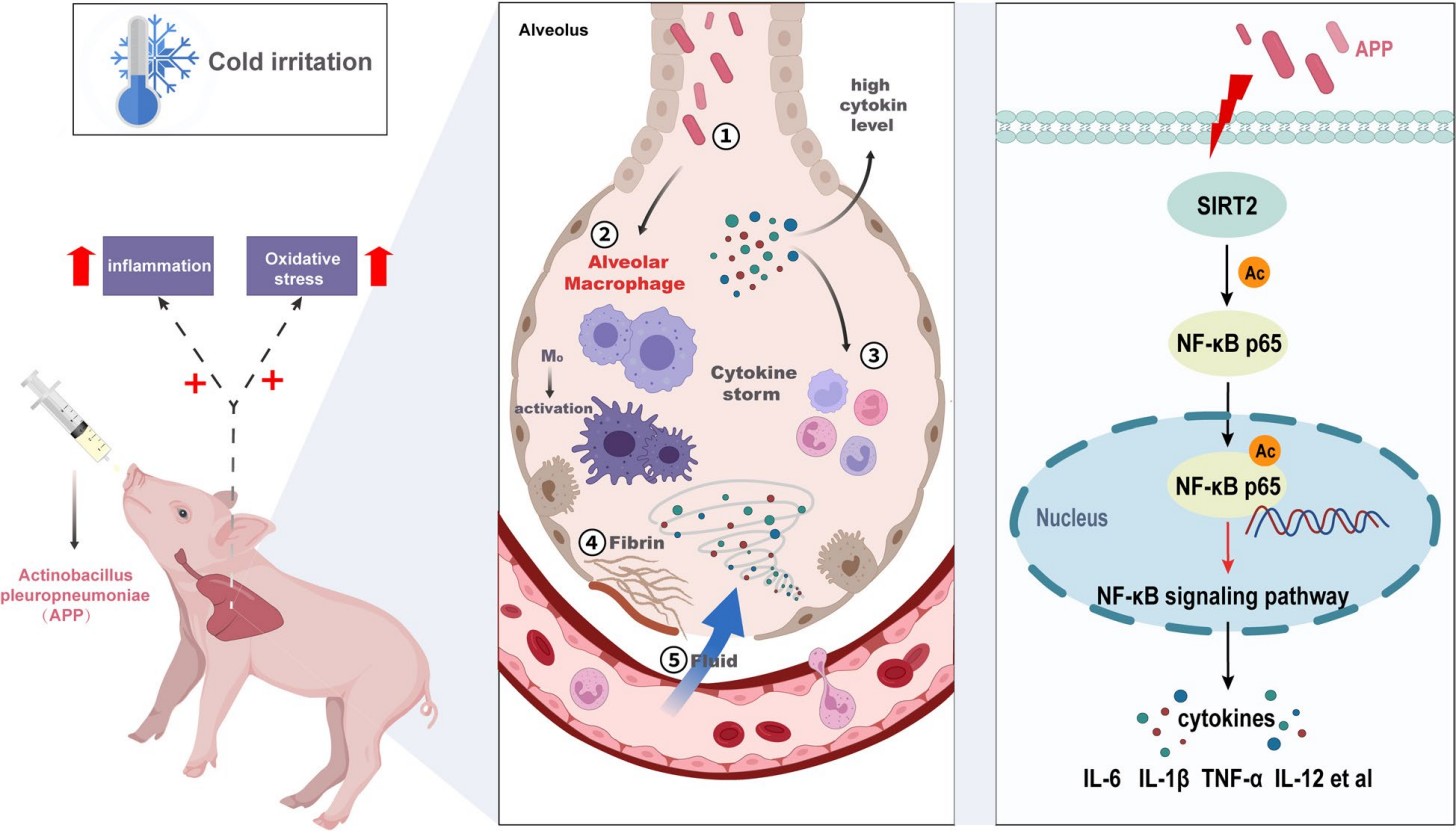
**SIRT2 participates in regulating the immune response of PAMs infected with APP under cold stimulation.** Inflammatory response and oxidative stress in the lungs of piglets were enhanced following APP infection under cold-stimulated conditions; AM activation and the cascade of synergistic effects between cytokines led to a “cytokine storm”; SIRT2 mediates the immune response of PAMs following APP infection by regulating NF-κB p65 acetylation modification. AM: alveolar macrophages; APP: *Actinobacillus pleuropneumoniae*; PAMs: porcine alveolar macrophages; SIRT2: sirtuin 2.

We show that cold-stimulated APP infection induces metabolic reprogramming of lung immune cells, thereby interfering with lung immune function. Furthermore, SIRT2 mediates the immune response of PAMs following APP infection by regulating NF-κB p65 acetylation modification (Figure 8). The results of this study provide a theoretical foundation and potential intervention targets for overcoming bottlenecks in the prevention and control of winter PRDC, thereby supporting future strategies to improve the safety and stability of the swine industry.

## Data Availability

The data and materials will be made available upon reasonable request.
